# On the Value of Quinine as a Therapeutic Agent

**Published:** 1857-04

**Authors:** Montrose A. Pallen

**Affiliations:** St. Louis


*On the Value of Quinine as a Therapeutic Agent.* By MONTROSE A. PALLEN, M. D., of St. Louis.

There is a tendency in the human mind to ascribe causation to mere antecedents—to presume that certain things bear the relation of cause and effect, when the true relation is antecedence and sequence only. From this common error medical science is by no means free. Disease departs from the human system, and the cure is ascribed to the remedies employed, when the true solution lies in the efforts of Nature. This is manifest enough when we reflect on the number of patients recovering under the ridiculous infinitesimal doses of the disciples of the Homœopathic practice.

This truth should always be present to the mind of the scientific physician. It inculcates on him the necessity of watching the operations of Nature, and of determining how much she is competent to cast off the elements of disease. Knowing the extent of her powers, we are prepared to assist her when she falters, and even, if necessary, to strengthen her well-directed endeavors.

There is also a tendency in the human mind to grasp with avidity any thing positive. Sciences which lead to certain results, are always pursued with energy. Hence, the charm, which the study of mathematics possesses for many persons. Hence the desire of all physicians to master the subject of auscultation and percussion in the exploration of visceral diseases. Hence the study of the microscope, and the use of the speculum. In a word, to this desire for certainty we ascribe the anxiety of the physician to listen for sounds which convey correct information, and to inspect diseased organs for occular demonstration. So too, in therapeutics, he seizes on a remedy which leads to known curative results; for this reason, quinine, with its well earned reputation in intermittent diseases, becomes a powerful and popular remedy for good and for evil. The physician, knowing its virtues wishes to enlarge its sphere of action—he administers it in various affections: the patient recovers, and that which is a mere sequence, becomes in his mind an effect.

We lay down these propositions as the result of our observations:

A. Quinine is an efficient remedy in intermittent diseases.

B. Many of these diseases require previous medication before it is proper to give quinine.

C. In continued diseases, whether they be fevers or inflammatory affections, quinine is of no value, except at their close, when a tonic is desirable, and then it is no better than many other vegetable bitters.

We need not insist on the value of quinine in intermittent and remittent fevers, and in intermittent neuralgia. In many such diseases nothing else is required; but it also happens, that when the large flabby and coated tongue denotes that there are elements in the system which ought to be thrown off, it is necessary that free purgation should first be resorted to: the condion of the tongue and the character of the alvine dejections affording the means of judging how far this is to be carried before quinine ought to be administered.

Quinine has its value in intermittent dysentery. In the fall season, when dysentery has both an inflammatory and a paludal element in its pathology, distinct intermissions become apparent. In this form of disease, the liver is much involved, and the indications are to restore the normal healthy actions of this organ, to subdue the colitis and to break up the exacerbations. Quinine alone is not apt to succeed—calomel and opium are slow of success; but a combination of each is attended with the happiest results. We do not mean that it is always proper to give them combined; it is better, first, to relieve the inflammatory element with calomel and opium, and then to give the quinine alone, or with opium if the latter be required to restrain the irritability of the bowels. Quinine has its value in intermittent pneumonia, an affection by no means of rare occurrence in this locality, and to be distinguished from ordinary pneumonia by the following characteristics: The rigor which ushers it in is more violent and longer continued, and is followed by intense heat, which, in a few hours, is succeeded by abundant perspiration. During this period there is great headache and sometimes intense lumbar pain; the pulse is rapid and soft, and not full and strong as in ordinary pneumonia.

Dr. Constant, practicing in the paludal districts of the department of Lôt, in France, says of this disease, that "there is never any purulent expectoration, these pneumonias never passing beyond the second stage, *i. e.*, red hepatization, the pulmonary engorgement being rather a sanguineous congestion than inflammation. Auscultation and percussion being of the highest value, often revealing the disease when unsuspected, a distinctive feature is the rapid passage from the first to the second stage of the disease, so that eight or ten hours after auscultation had revealed only a circumscribed *râle*, a whole side will be found hepatized. Under the influence of large doses of quinia this rapidly disappears, giving way to returning subcrepitant *râle* during the remission of the fever, but returning again during the paroxysm, if this have not been cut short. The crepitant *râle* of the first stage is almost always moist, the *parchment-cracking râle* only having been heard for a short period, two or three times in more than sixty cases. It invades large surfaces rapidly, being heard posteriorly, sometimes laterally, but never in front. This form of pneumonia especially affects the posterior parts of the lower lobes. It especially appears in summer and autumn, while ordinary pneumonia prevails in spring and winter. It attacks all ages indiscriminately, except early infancy. The blood which flows from a vein is often below the normal temperature, very black and deficient in plasticity. After rest, its surface acquires a blueish color, especially if the patient is taking quinia. The clot is slow of formation and soft. The buffy coat is absent or very thin, and inclines to a blueish color. The condition of the blood, conjoined with the soft pulse and red hepatization, constitutes the chief distinctive sign of the affection.

"In this district, during winter, purely inflammatory pneumonia is met with; but in proportion to the high temperature and the production of malarial emanations, this inflammatory element is replaced by the paludal one. There are, indeed three forms met with. 1st. Simple pneumonia. 2d. Spring inflammatory pneumonia, complicated with intermittent paroxysm. 3d. Summer and autumn intermittent pneumonia. The first requires bleeding and antimony; the second, antiphlogistic treatment with quinia, given either simultaneously or subsequently; and the third, quinia in combination with external revulsives. These forms may still undergo further admixture, according as the inflammatory or paludal element prevails, requiring appropriate modification in the treatment."

In dysenteries of a continued type, that is, when they are merely inflammations, quinine is useless. In pure inflammatory pneumonia, no man in his senses ought to prescribe it. If, then, Quinine be valuable in these affections when they are of an intermittant type, that is to say, when they have a paludal element mixed with them, and it be pernicious when they are purely inflammatory and continuous, would not analogy teach us that the same result would follow in fevers? Paludal fevers are remittent and intermittent. Is such the case with typhus and typhoid fevers? We must not confound, as a slight abatement of the frequency of the pulse, a distinct remission. This is observed in many affections after the repose and quietude of night. If, however, we exclude the argument by analogy, experience will teach us. We have seen many cases of typhoid and typhus in our hospitals, in which the quinine treatment has been totally discarded, the rates of mortality decreasing in a greater proportion than they increased under its treatment. According to Dr. E. S. Smith, of this city, who has had ample opportunities to investigate these forms of fever both at home and abroad, the results of his experience are, that in the pure forms of typhus and typhoid, there being no paludal element, the administration of quinine is never attended with beneficial results. Typhoid fever, whose characteristic lesion is ulceration of the bowels, seems to us, from statistics carefully gathered to be made worse by quinine, as the diarrhœa was universally made worse, the head symptoms increased, and the patient more prostrated after the administration than before. In this connection we will quote the words of Dr. Barclay, in the London *Medical Gazett & Times*, January, 1853. After giving the reports of the cases treated by him at St. George's Hospital, some with quinine, and some without quinine, he says:

"I must here distinctly state, that when I commenced this report I had no idea what the result would be, and, so far from believing it unfavorable, had hoped, that, excluding some unfortunate cases, the treatment of fever with quinine would prove rather more speedy, safe, and effectual, than by ordinary modes. I am sorry to be convinced that it has no advantages. It may be well to state in conclusion, that the prevalent type of fever has been what would be called 'typhoid,' not true 'typhus;' one or two had the aspect of congestive typhus, but wanted the purple-motted rash."—*St. Louis Medical & Surgical Journal.*

